# Patients’ Outcome Expectations Matter in Psychological Interventions for Patients with Diabetes and Comorbid Depressive Symptoms

**DOI:** 10.1007/s10608-014-9667-z

**Published:** 2015-01-18

**Authors:** Evelien Snippe, Maya J. Schroevers, K. Annika Tovote, Robbert Sanderman, Paul M. G. Emmelkamp, Joke Fleer

**Affiliations:** 1 Department of Health Psychology, University Medical Center Groningen, University of Groningen, P.O. Box 196, 9700 AD Groningen, The Netherlands; 2 Department of Psychology, Health and Technology, University of Twente, Enschede, The Netherlands; 3 Department of Clinical Psychology, University of Amsterdam, Amsterdam, The Netherlands; 4 The Center for Social and Humanities Research, King Abdul Aziz University, Jeddah, Saudi Arabia

**Keywords:** Expectations, Diabetes, Homework, Mindfulness, Depression

## Abstract

This study examined whether patients’ expectations of treatment outcome predict treatment completion, homework compliance, and depressive symptom improvement in cognitive behavior therapy (CBT) and mindfulness-based cognitive therapy (MBCT). Study participants were patients with diabetes and comorbid depressive symptoms who were randomized to 8 sessions of either CBT (n = 45) or MBCT (n = 46), both individually delivered. The results showed that high outcome expectations were predictive of post-treatment depressive symptoms in CBT and MBCT, but not of early and mid-treatment symptoms. Patients’ outcome expectations predicted treatment completion in CBT and MBCT as well as homework compliance in MBCT. Homework compliance did not mediate the association between patients’ outcome expectations and post-treatment depressive symptom improvement. The findings do not support the hypothesis that patients’ expectations have an immediate impact on patients’ mental state and partially support the notion that patients are less involved in treatment when they hold low expectations for improvement.

## Introduction

The prevalence of depressive symptoms in patients with diabetes is almost twice as high as in individuals without a chronic disease (Roy and Lloyd 2012). Fortunately, there are psychological interventions available that have been shown to be efficacious in reducing depressive symptoms in patients with diabetes, such as cognitive behavior therapy (CBT; Gonzalez et al. 2010; Lamers et al. 2010; Lustman et al. 1998; Penckofer et al. 2012; van Bastelaar et al. 2011) and mindfulness-based cognitive therapy (MBCT; Schroevers et al. 2013; van Son et al. 2013). Yet, not all patients with diabetes and depressive symptoms show a clinically significant improvement in response to these interventions (see e.g., Penckofer et al. 2012; van Son et al. 2013). In addition, between 15 and 20 % of the patients has been found to drop out of CBT and MBCT (Beltman et al. 2010; Kingston et al. 2007) and not all individuals comply with treatment procedures including home practice, which is of importance to acquire skills in both CBT (Beck et al. 1979; MBCT Segal et al.^2002^). A next step is therefore to investigate factors that possibly contribute to compliance, treatment completion, and symptom improvement in order to know how to optimize the intervention process. In this study, the role of patients’ outcome expectations is examined concerning homework compliance, drop out from treatment, and depressive symptom improvement in individually delivered CBT and MBCT for patients with diabetes and comorbid depressive symptoms.

## Patients’ Outcome Expectations

In the 1960s and 1970s, it was already suggested that patients’ outcome expectations may be of importance in understanding the efficacy of psychotherapy (Frank 1968; Goldstein 1960) and CBT (Emmelkamp 1975). Patients’ outcome expectations refer to patients’ beliefs about the consequences of receiving treatment (Constantino et al. 2011), such as the belief that treatment will lead to improvement. A meta-analysis of Constantino et al. (2011) showed a small but consistent positive association between patients’ outcome expectations and outcomes of psychological treatments. The few studies that specifically focused on CBT also showed that higher outcome expectations are predictive of post-treatment depressive symptom reduction (Meyer et al. 2002; Sotsky et al. 1991; Webb et al. 2013). Early expectancy theorists posed that treatment outcomes may be positively affected by the creation of hope induced by patients’ expectations of improvement (Frank 1973; Goldstein 1960). Thus, high outcome expectations may directly affect patients’ mental state and as such leads to rapid symptom relieve (Frank 1968). Such early gains may be important for clinical outcomes of CBT as it has been found that significant depressive symptom reduction can occur in the first few sessions of CBT and that such early gains have a moderate effect on treatment outcome, even at follow-up (Aderka et al. 2012). To our knowledge, no studies examined whether patients’ outcome expectations are related to depressive symptom improvement early in treatment.

Besides an immediate effect on depressive symptoms, patients’ outcome expectations may also indirectly affect treatment efficacy by their impact on patients’ behavior during treatment. In particular, it has been posed that outcome expectations can influence patients’ engagement and involvement in therapy (see e.g., Ilardi and Craighead 1994; Meyer et al. 2002; Webb et al. 2013). This assumption is based on the general idea that high expectations may induce persistent effort to achieve a goal whereas low expectations may result in disengagement from desired goals (Austin and Vancouver 1996; Carver and Scheier 1998). If patients do not expect to improve in response to treatment, they may disengage from their goal to participate in treatment and as a result drop out of treatment. In depression research, this was evidenced by two studies showing that patients’ expectations predicted treatment dropout in CBT for anxiety and depression (Cavanagh et al. 2009) and in CBT for major depression and dysthymia (Schindler et al. 2013).

Assuming that patients’ expectations influence engagement in treatment, these outcome beliefs may not only affect treatment completion but also compliance with treatment procedures (Lick and Bootzin 1975), such as homework assignments (Westra et al. 2007). When patients expect that they will improve as a result of their treatment, they may increase their effort to make their treatment work (Greenberg et al. 2006) and thus perform more homework assignments (Detweiler and Whisman 1999). Home practice is assumed to influence the effectiveness of both CBT and MBCT, as homework may teach patients the necessary skills to cope with depressive symptoms (Beck et al. 1979; Segal et al. 2002). Thus, homework compliance may in fact mediate the association between patients’ outcome expectations and treatment outcomes. Currently, only a few studies investigated the association between patients’ outcome expectations and homework compliance, with no studies in psychological treatments for depressive symptoms. One study on CBT for anxiety disorders did not find an association between patients’ outcome expectations and homework compliance (LeBeau et al. 2013), whereas two studies showed that positive outcome expectations predicted homework compliance in CBT for anxiety (Westra et al. 2007) and in CBT for obsessive compulsive disorder (Lewin et al. 2011). Westra et al. (2007) also showed that homework compliance mediated the association between patients’ expectations for anxiety change and actual symptoms change. To draw more firm conclusions on the role of outcome expectations in patients’ behavioral involvement in treatment, more research is warranted.

## Current Study

It is yet unclear if and how patients’ outcome expectations affect the treatment process and depressive symptom improvement in psychological treatments for patients with diabetes, as no studies have investigated this. In CBT for chronic somatic diseases, only few studies examined patients’ outcome expectations and found that these beliefs predicted the outcomes of CBT for chronic pain (Goossens et al. 2005; Smeets et al. 2008) and chronic fatigue (Heins et al. 2013). To our knowledge, no studies examined the role of outcome expectations in MBCT. Examining the role of patients’ expectations in depressive symptom improvement and homework compliance might in particular be relevant in patients with diabetes, because it is known that treatment non-compliance is high in patients with diabetes and comorbid depressive symptoms (Gonzalez et al. 2008).

The primary aim of the present study is to investigate whether patients’ outcome expectations predict dropout rates, homework compliance, and depressive symptom improvement in individually delivered CBT and MBCT for patients with diabetes and comorbid depressive symptoms. Secondary aims of the study are to examine the association between homework compliance and depressive symptom improvement and to examine whether the association between outcome expectations and depressive symptom improvement is mediated by homework compliance. Based on the early works of expectancy theorists, it is hypothesized that higher outcome expectations predict rapid depressive symptom improvement in both CBT and MBCT. Outcome expectations are also hypothesized to predict post-treatment depressive symptoms. Assuming that patients’ expectations for improvement predispose patients to engage in treatment procedures, it is hypothesized that lower outcome expectations predict higher dropout rates, poorer compliance with homework assignments and a decrease in homework compliance over the course of CBT and MBCT. Finally, it is expected that compliance with homework assignments predicts depressive symptom improvement both during treatment and at post-treatment in CBT and MBCT as home practice is assumed to contribute to the development of skills that enable coping with depressive symptoms. As both outcome expectations and homework compliance are expected to predict treatment outcomes and since outcome expectations are hypothesized to predict homework compliance, it is also expected that the association between expectations and post-treatment depressive symptom improvement is mediated by homework compliance.

## Methods

The current study is embedded in a multi-center randomized controlled trial on the efficacy of CBT and MBCT for depressive symptoms in patients with diabetes. The study was approved by the Medical Ethical Committee of the University Medical Center Groningen. All procedures followed were in accordance with the ethical standards of the Medical Ethical Committee of the University Medical Center Groningen and with the Helsinki Declaration of 1975, as revised in 2000. All participants provided informed consent for being included in the study. The design of the study as well as a full report of the recruitment of patients along with the flowchart and the primary outcomes are reported elsewhere (Tovote et al. 2013, 2014). The results of the trial showed that both CBT and MBCT are efficacious in reducing depressive symptoms in patients with diabetes in comparison with a waiting list control condition (Tovote et al. 2013, 2014). Below, an abstract of the methods is described.

## Participants

Most participants were recruited through a consecutive screening procedure at four hospitals in the Netherlands between June 2011 and February 2013. A few patients were referred by a physician or were self-referred. Inclusion criteria were a Beck depression inventory-II (BDI-II) score ≥14, a diagnosis of diabetes (type 1 or type 2) ≥3 months, and age between 18 and 70. Exclusion criteria were inability to read and write, pregnancy, severe psychiatric comorbidity, acute suicidal ideations, receiving psychological treatment within 2 months prior to inclusion, and unstable use of antidepressants within 2 months prior to inclusion. Eligible participants who provided written informed consent were randomized to immediate CBT, immediate MBCT or a 3 months waiting list control condition. After 3 months, participants in the waiting list control condition were randomized for a second time to either CBT or MBCT. Participants were informed that treatment would start within 3 months and that they would be randomized to one out of two psychological treatments that focus on reducing depressive thoughts and feelings. No specific information was provided on the type of intervention.

In the current study, data are used of patients who received CBT or MBCT either directly or after a waiting period of 3 months. Concerning the waiting list condition, only the data were used of those participants who still reported at least mild depressive symptoms (BDI-II ≥14) after the waiting list period. In the original trial, 94 patients gave consent to participate and were randomized to CBT (N = 32), MBCT (N = 31), or the waiting list control condition (CBT: N = 15, MBCT: N = 16). Three participants did not report at least mild depressive symptoms after the waiting period and were therefore excluded from the current study. The total sample used in the current study consisted of 91 participants who received either MBCT (n = 46) or CBT (n = 45).

## Cognitive Behavior Therapy (CBT)

Patients received individual CBT based on CT as developed by Beck et al. (1979). The treatment was shortened to 8 weekly sessions of 45–60 min. The sessions and homework exercises included activity monitoring, scheduling and performing pleasant or functional activities, identifying and challenging dysfunctional thoughts, and relapse prevention. The number of assigned homework exercises was personalized. Patients were asked to perform homework exercises for a maximum of half an hour a day, consisting of 1–2 homework exercises a day.

## Mindfulness-Based Cognitive Therapy (MBCT)

Patients received individually delivered MBCT based on the standardized group MBCT manual developed by Segal et al. (2002). The duration of the original exercises and the inquiry was shortened to make the program fit in 8 weekly sessions of 45–60 min (for a detailed description of the individual MBCT manual see Schroevers et al. 2013). The MBCT sessions and homework exercises included formal mindfulness exercises (i.e., guided meditation/yoga such as the body-scan or mindful stretching), informal exercises (e.g., 3-min breathing space, mindfulness of a routine activity) and CBT exercises (e.g., pleasant events calendar, relapse prevention). Patients were asked to perform homework exercises for approximately 30–45 min a day, consisting on average of 1 formal exercise, 1–4 informal exercises and 1 CBT exercise a day.

## Therapists and Training

Therapists were nested within type of treatment to enhance treatment differentiation. Twelve therapists delivered CBT (male *N* = 2) and nine therapists delivered MBCT (male *N* = 1). All therapists finished at least their Master’s degree in Clinical Psychology and had received clinical training. The MBCT therapists were all experienced in mindfulness practice and had participated in a mindfulness-based treatment as a participant. Of all therapists, seven CBT therapists and five MBCT therapists had fewer than 3 years of experience in CBT or MBCT. These therapists received 2 days of training in CBT or MBCT which mainly entailed role playing. All CBT and MBCT therapists received a structured treatment manual including information on diabetes and depression as well as specific instructions on exercises, inquiry, and homework assignments per session. All therapists received supervision once every three weeks. The CBT training and supervision was provided by the fifth author who is a licensed clinical psychologist and CBT therapist with more than 35 years of experience in providing CBT supervision. The MBCT training and supervision was provided by the second author; a mental health psychologist who received extensive training in MBSR/MBCT and has provided more than 25 mindfulness programs in the past 7 years. Therapists provided treatment to a minimum of 2 patients and a maximum of 8 patients, with a median of 4 treated patients per therapist. Adherence to the treatment manual was sufficient both in MBCT (86 %) and in CBT (79 %). Adherence represents the average percentage of adopted prescribed treatment techniques during the second and sixth treatment session as rated independently by two out of three trained students pursuing a Master’s degree in Clinical Psychology. The overall agreement between the raters was 85.7 % in CBT and 94.3 % in MBCT.

## Measures

### Outcome Expectations

Patients’ expectations for improvement were assessed with a 2-item questionnaire based the work of Borkovec and Nau (1972) and the expectancy subscale of the credibility/expectancy questionnaire (Devilly and Borkovec 2000). The first question was: “How would you estimate the likelihood that his treatment will help you?” The VAS response scale of the first question ranged from “unlikely” (0) till “for sure” (10). The second question was: “By the end of the treatment period, how do you expect you will feel?” The VAS response ranged from “worse” (0) till “completely recovered” (10). The mean score based on the two items may range from 0 (low outcome expectations) till 10 (high outcome expectations). The internal consistency of the scale was sufficient (α = 0.75, r = 0.62). As patients were informed properly on the specific treatment approach during the first session, patients’ outcome expectations were assessed after the first treatment session.

### Depressive Symptoms

Depressive symptoms were assessed with the Beck depression inventory-II (Beck et al. 1996). The BDI-II is a 21 item self-report measure that assesses severity of depressive symptomatology with a total score ranging from 0 to 63. In the current study, the internal consistency of the BDI-II was sufficient (α ranging between 0.83 and 0.93). The BDI-II was administered at pre-treatment (at baseline or after the waiting list period), after the second treatment session, after the fourth session treatment and after the eight session (post-treatment).

### Dropout

Participants who received at least 6 sessions (3/4 of treatment) were considered to have received an appropriate dose of treatment (Tovote et al. 2014). Yet, attending the full 8 sessions is expected to be most beneficial, and therefore treatment completion was used as a second measure of dropout. Thus, dropout was measured in two ways: dropout before having received 6 sessions and dropout before having received 8 sessions.

### Homework Compliance

At the end of each treatment session, participants received a record form with the assigned homework exercises for the coming week. The completed forms were returned at the start of each following treatment session. The forms were specific to treatment and session because different homework exercises were assigned in CBT and MBCT and because the assigned homework differed per session. Each day, participants ticked boxes with homework exercises that they performed on that particular day. In MBCT, participants were also asked to record daily time in minutes spend on formal meditation exercises.

Homework compliance was measured in one way in CBT and in three ways in MBCT. For both CBT and MBCT, the number of weekly performed homework exercises was used. For MBCT, also a weighted percentage of formal, informal, and CBT exercises was used as a second measure of homework compliance, because these MBCT exercises differ in their duration. To calculate this measure, the proportion of performed exercises per week was computed for formal, informal, and CBT exercises separately. The average of these proportions, with a maximum of 1, was multiplied by 100. In addition to that, the amount of time spent on formal meditation practice per week was used as a third measure of homework compliance in MBCT. As the duration of the exercises in CBT are similar, other measures of homework compliance were not computed in CBT.

### Statistical Analyses

Logistic regression analyses were conducted in SPSS 20.0 to examine patients’ outcome expectations as a predictor of treatment dropout. Because the sample of dropouts is small, the data of CBT and MBCT were pooled for the analyses concerning dropout.

Multilevel analyses were performed using STATA XTmixed. In a first model, it was examined if patients’ outcome expectations were predictive of homework compliance. The analyses were run with homework compliance assessed at session 1 till session 6 (level 1) nested within individuals (level 2). Outcome expectations and a variable denoting time (Bolger and Laurenceau 2013) were included as predictors. In a second model, an interaction between expectations and time was included as an additional predictor to examine whether lower outcome expectations would predict a decrease in homework over time.

A third multilevel analysis was performed to examine patients’ outcome expectations as a predictor of depressive symptom improvement. Depressive symptoms (at pre-treatment, after session 2, after session 4, and at post-treatment) were included at level 1 and individuals at level 2. Expectations were used to predict depressive symptoms at session 2, session 4, and at post-treatment. To do so, three dummy variables for time and interactions between the time dummies and patients’ expectations were included. The time dummies reflect the change in depressive symptoms from pre-treatment to session 2, to session 4, and to post-treatment. The interactions between the time dummies and patients’ expectations reflect the associations between expectations and depressive symptoms at the specific time points.

A fourth multilevel analysis was performed to examine if homework compliance predicted subsequent depressive symptom improvement. Depressive symptoms at pre-treatment, after session 2, after session 4, and at post-treatment were included at level 1 and individuals at level 2. Homework compliance after session 1 was used to predict depressive symptoms at session 2, average homework compliance from session 1 till 3 was used to predict depressive symptoms after session 4, and average homework compliance from session 1 till 6 was used to predict depressive symptoms at post-treatment.

All multilevel analyses were conducted according to the intention-to-treat approach. The data of several patients were partially missing for some analyses because they dropped out of treatment prematurely. The advantage of using multilevel analyses is that missing data are handled by using all available data, including cases with partially missing data (Snijders and Bosker 2012). Rerunning the multilevel models with therapists as a third level (i.e., patients nested within therapists) either did not concave or did not improve model fit according to the akaike information criterion (AIC); AIC values increased with 2 points when therapists were included as a third level.

Since including therapists as a third level did not change the results either, the final models were run without therapists as a third level.

Finally, it was explored whether the association between patients’ outcome expectations and post-treatment depressive symptoms improvement was mediated by homework compliance. This mediation model was tested with the macro process for SPSS (Hayes 2013) based on the Bootstrap procedure of Preacher and Hayes (2004). This approach provides an estimate and 95 % confidence interval of the indirect effect (*ab*) (i.e., the product of the path from the independent variable to the mediator and the path from the mediator to the dependent variable; Preacher and Hayes 2004), based on 10,000 Bootstraps. Post-treatment depressive symptoms were included as the outcome, patients’ outcome expectations as the independent variable, average homework compliance from session 1 till session 6 as the mediator, and pre-treatment depressive symptoms as a covariate. The mediation analyses are considered exploratory since the power to find a significant mediation effect is small due to the relatively small sample size and missing data.

## Results

### Preliminary Analyses

Characteristics of the study variables are shown in Table 1. In both CBT and MBCT, participants’ outcome expectations were on average at the middle of a VAS scale ranging from 0 (low outcome expectations) till 10 (high outcome expectations). Drop-out rates in CBT and MBCT were comparable; approximately one-fourth of the participants completed fewer than 6 sessions and approximately one-third of participants did not complete the full treatment program. In MBCT, participants performed on average 2 homework exercises a day, they completed on average more than half of the assigned formal and informal exercises, and spent on average 2 h on formal mindfulness practice per week. In CBT, participants performed on average 1.4 homework exercises a day. The average number of performed exercises per week was lower in CBT than in MBCT since fewer homework exercises are assigned in CBT in comparison with MBCT.

**Table 1:** Characteristics of the study variables

	CBT	MBCT
	M (SD)/%	M (SD)/%
Outcome expectations	5.3 (1.5)	5.8 (1.1)
Depressive symptoms		
Pre-treatment	24.7 (8.3)	24.1 (8.3)
Session 2	22.8 (7.0)	19.5 (8.1)
Session 4	21.8 (8.9)	18.4 (10.5)
Post-treatment	17.3 (11.0)	17.2 (10.7)
Weekly homework compliance		
Number	9.7 (4.0)	15.3 (7.0)
Percentage formal/informal		61.6 (23.3)
Minutes		124.8 (61.4)
Treatment completion		
Sessions 1–5	27 %	26 %
Sessions 6–7	9 %	5 %
Session 8	64 %	69 %

Depressive symptoms were assessed with the BDI-II. Homework compliance = average homework compliance from session 1 up to session 6

### Are Outcome Expectations Predictive of Dropout?

When pooling the data of CBT and MBCT, outcome expectations did not significantly predict dropout before session 6 (χ^2^ = 1.95, *p* = 0.16, odds ratio (OR) 0.74, 95 % CI 0.48–1.13), but they did significantly predict dropout before the last (eighth) treatment session (χ^2^ = 6.02, *p* = 0.01, OR 0.60, 95 % CI 0.38–0.93). Thus, the odds of dropping out before the last treatment session is lower when participants have higher outcome expectations. Although underpowered, dropout rates were explored for CBT and MBCT separately as well. Outcome expectations were predictive of drop-out before the last session in CBT (χ^2^ = 3.76, *p* = 0.05, OR 0.60, 95 % CI 0.34–1.05) and in MBCT at borderline significance (χ^2^ = 2.89, *p* = 0.09, OR 0.54, 95 % CI 0.25–1.15).

### Are Outcome Expectations Predictive of Homework Compliance?

Higher outcome expectations predicted performance of a higher number of homework exercises in MBCT, but not in CBT (see Model 1, Table 2). In MBCT, higher outcome expectations also significantly predicted a higher weighted percentage of formal, informal and CBT exercises (*B* = 15.59, *SE* = 3.43, *p* < 0.01), and more time spent on formal meditation practice per week (*B* = 28.86, *SE* = 9.47, *p* < 0.01). There was a significant interaction between patients’ outcome expectations and time when predicting the number of performed homework exercises in MBCT, but not in CBT (see Model 2, Table 2). This indicates that higher outcome expectations predicted an increase in the number of performed homework exercises over the course of MBCT. Patients’ outcome expectations did not predict an increase over time in the weighted percentage of formal, informal and CBT exercises (*B* = −0.65, *SE* = 0.94, *p* = 0.49) and the time spent on formal meditation practice (*B* = 0.61, *SE* = 2.31, *p* = 0.79) in MBCT.

**Table 2:** Predicting number of performed homework exercises by patients’ expectations

	MBCT		CBT	
	Model 1	Model 2	Model 1	Model 2
	*B (SE)*	*B (SE)*	*B (SE)*	*B (SE)*
Fixed effects				
Intercept	10.3 (1.0)**	10.6 (1.0)**	8.8 (0.6)**	8.8 (0.6)**
Time	1.0 (0.3)**	0.7 (0.3)*	0.4 (0.3)	0.4 (0.3)
Expectancies	4.1 (0.8)**	3.1 (1.0)**	−0.4 (0.3)	−0.2 (0.4)
Time expect*		0.7 (0.3)*		−0.2 (0.2)
Random effect variances				
Intercept	13.4 (5.8)**	14.5 (6.0)**	<0.1 (<0.1)	<0.1 (<0.1)
Time	1.2 (0.7)*	0.9 (0.6)	2.0 (0.7)**	2.1 (0.7)**
Residual	27.9 (3.5)**	27.7 (3.4)**	16.3 (2.3)**	16.1 (2.2)**
*N/obs*	34/176	34/176	38/192	38/192

*N* number of participants, *obs* number of observations

* *p* < 0.05; ** *p* < 0.01

### Are Outcome Expectations Predictive of Depressive Symptom Reduction?

As hypothesized, higher outcome expectations predicted lower levels of depressive symptoms after treatment (i.e., after session 8), both in CBT and in MBCT (see Model 3 in Table 3). In both treatments, patients’ outcome expectations did not predict depressive symptom change early in treatment, neither after the second session nor after the fourth session.

**Table 3:** Predicting depressive symptoms by patients’ expectations and homework compliance

	MBCT		CBT	
	Model 3	Model 4	Model 3	Model 4
	*B (SE)*	*B (SE)*	*B (SE)*	*B (SE)*
Fixed effects				
Intercept	22.8 (1.4)**	24.0 (1.5)**	24.2 (1.3)**	24.4 (1.3)**
∆ session 2	−3.6 (0.9)**	3.9 (0.9)**	−1.1 (0.9)	−0.9 (0.9)
∆ session 4	−4.6 (1.1)**	−4.7 (1.1)**	−3.1 (1.2)**	−2.8 (1.2)*
∆ session 8	−5.7 (1.0)**	−6.5 (1.2)**	−8.4 (1.2)**	−8.5 (1.3)**
Expect*session 2	−0.7 (0.9)		0.1 (0.6)	
Expect*session 4	−0.2 (1.0)		−0.4 (0.7)	
Expect*session 8	−3.0 (1.0)**		−2.2 (0.7)**	
Homework*session 2		−0.1 (0.1)		−0.1 (0.3)
Homework*session 4		−0.2 (0.2)		−0.1 (0.3)
Homework*session 8		−0.2 (0.1)		−0.1 (0.3)
Random effect variances				
Intercept	52.8 (14.3)**	63.0 (17.3)**	33.4 (14.8)**	32.0 (17.8)
Residual	18.7 (4.6)**	22.3 (6.1)**	29.8 (10.9)**	35.6 (14.9)**
*N/obs*	38/133	39/134	38/143	40/141

expect*session = association between patients’ outcome expectations and depressive symptoms at session, homework*session = association between previous homework compliance and depressive symptoms at session

*N* number of participants, *obs* number of observations

* *p* < 0.05; ** *p* < 0.01

### Is Homework Compliance Predictive of Depressive Symptom Reduction?

Contrary to our hypothesis, the average number of performed homework exercises per week did not predict change in depressive symptoms early in treatment or after treatment, neither in CT, nor in MBCT (see Model 4 in Table 3). Similarly, depressive symptoms after receiving MBCT were neither predicted by the weighted percentage of formal, informal, and CBT exercises (*B* = −0.08, *SE* = 0.05, *p* = 0.09), nor by the amount of time spent on formal exercises (*B* = −0.01, *SE* = 0.02, *p* = 0.50).

### Does Homework Mediate the Association Between Outcome Expectations and Depressive Symptoms?

Patients’ outcome expectations did not predict post-treatment depressive symptom improvement indirectly through a higher number of performed homework exercises, neither in CBT (*ab* = 0.01; 95 % CI = −0.44 to 0.63), nor in MBCT (*ab* = 0.35; 95 % CI = −1.46 to 2.61). In MBCT, the association between outcome expectations and post-treatment depressive symptom reduction was also not mediated by the weighted percentage of formal, informal, and CBT exercises (*ab* = −0.05; 95 % CI = 1.85–2.20), nor by the amount of time spent on formal exercises (*ab* = 0.94; 95 % CI = −0.90 to 3.19).

## Discussion

The primary aim of the present study was to investigate whether patients’ outcome expectations contribute to patients’ engagement in treatment and treatment outcomes in individually delivered CBT and MBCT for patients with diabetes and comorbid depressive symptoms. The findings imply that patients are more inclined to complete CBT and MBCT and that they benefit more from these treatments when they have higher expectations of improvement. Such high outcome expectations also seem to positively affect compliance with homework in MBCT, but not in CBT. The results did not support the assumptions that homework compliance predicts depressive symptom improvement and that homework compliance mediates the association between patients’ outcome expectations and treatment outcomes, neither in CBT, nor in MBCT.

The current study extends previous findings by showing that outcome expectations are predictive of post-treatment depressive symptom improvement, not only in CBT, but also in MBCT for patients with diabetes and comorbid depressive symptoms. This finding supports the assumption that patients’ expectations are a ‘common factor’ of treatment effectiveness (Weinberger and Eig 1999), as these beliefs predict treatment outcomes independent of the type of treatment. This assumption is also supported by research indicating that higher expectations predict symptom improvement in different forms of CBT for a broad range of diagnoses (Chambless and Ollendick 2001; Lewin et al. 2011; Webb et al. 2013) as well as in psychological interventions targeting physical health related problems (e.g., Finch et al. 2005; Goossens et al. 2005).

Patients’ expectations could be a common factor because these beliefs might have an immediate effect on patients’ mental state and therefore lead to rapid symptom remission (Frank 1973; Goldstein 1960). However, the results did not support this assumption since no associations were found between patients’ expectations and depressive symptom improvement after the first few sessions, neither in CBT nor in MBCT. As patients’ expectations only predicted post-treatment depressive symptoms, it seems plausible that mediating processes are at play that takes a longer time to affect depressive symptoms.

Our findings support the notion that patients may disengage from treatment when they hold low expectations for improvement (Arnkoff et al. 2002). Low outcome expectations increased the odds of dropout before the last treatment session in both CBT and MBCT. However, patients’ expectations were not associated with dropout before the sixth treatment session. A likely explanation for this finding is that too few people dropped out before session six to be able to find an effect. The results regarding the role of expectations in treatment completion replicate the findings of Schindler et al. (2013) in CBT for depression. The current study expands their findings by showing that expectations not only affect dropout in CBT, but also in MBCT for depressive symptoms in patients with diabetes. In clinical practice, it might thus be relevant to raise patients’ expectations to increase the chance of treatment completion, for example, by promoting patients’ self-efficacy or providing a strong treatment rationale (Constantino et al. 2012). This might be in particular relevant for patients with diabetes who enroll in psychological interventions through screening for depressive symptoms, like in our study, as these patients probably have lower expectations than those who seek treatment themselves.

In addition, modest support was found for the assumption that patients increase their effort to make their treatment work when they expect to improve in response to treatment. The results showed that patients’ compliance with MBCT homework is poorer when they hold lower expectations for improvement. In CBT, there was no association between outcome expectations and homework compliance. This difference might be due to the fact that more daily homework is assigned in MBCT than in CBT. As the burden of homework is higher in MBCT, it could be that patients’ outcome expectations mainly influence the investment of more persistent effort. Another explanation might be that the number of performed homework assignments in CBT is not a good measure of compliance because the amount of assigned homework may vary between individuals.

A secondary aim of the present study was to examine the role of homework compliance in treatment efficacy. The findings indicate no association between homework compliance and depressive symptom improvement in CBT and MBCT. Only a tendency was found towards an effect of the weighted percentage of performed formal, informal and CBT exercises on post-MBCT depressive symptoms. A few previous studies also showed small or non-significant associations between homework compliance and depressive symptom improvement after receiving CBT (e.g., Burns and Nolen-Hoeksema 1991) and Mindfulness-Based Stress Reduction (Carmody and Baer 2008). The non-significant associations could be due to that almost all participants performed at least one homework assignment a week, which might already be sufficient to result in a decrease in depressive symptoms. Therefore, no firm conclusions can be drawn on whether homework performance contributes to the effects of CBT and MBCT on depressive symptoms. Furthermore, the amount of assigned homework might have varied in CBT according to the needs of a particular patient and willingness to complete assignments. This variation might also explain the non-significant association between number of performed exercises and treatment outcome in CBT. Another explanation for the null findings might be that completion of homework assignments is not a measurement of the quality of home practice and the extent to which patients incorporate the trained skills in their daily life, which may be more important for reducing depressive symptoms than the amount of performed homework (Schmidt and Woolaway-Bickel 2000). In our study, the effect of the total number of formal and informal mindfulness exercises was greater than the time spend on formal meditation exercises, which may be an indication for the relative importance of informal practice in MBCT.

The absence of an association between homework compliance and depressive symptom improvement might also explain why homework compliance did not mediate the association between patients’ outcome expectations and post-treatment depressive symptom improvement. Also, the power to find a significant mediation effect was small in the current study because of missing data in the mediation analyses. A sufficiently powered study on CBT for anxiety did find that homework compliance mediated the association between expectancy for change and symptoms improvement (Westra et al. 2007). Furthermore, Westra et al. (2007) used a different method to assess homework compliance. They asked patients once to rate the amount, time, and effort they spent on homework on a 5-point Likert scale (*none* to *a whole*) after the second session. This item might show stronger associations with patients’ expectations as it captures perceived effort instead of the specific type and number of performed homework exercises.

Although the association between patients’ expectations and post-treatment symptom change could not be explained by homework compliance in the present study, previous studies suggest that other indicators of patients’ behavioral involvement may be candidate mediators of the expectancy-outcome association. For example, Webb et al. (2013) showed that the use and acquisition of CBT skills mediated the association between patients’ outcome expectations and symptom improvement in CBT for major depression. The idea that patients’ expectations affect patients’ behavior is also evidenced by studies outside the field of psychological interventions showing that higher outcome expectations are associated with more exercise behavior in endometrial cancer survivors (Basen-Engquist et al. 2013) and patients with COPD (Kaplan et al. 1984). Future research could provide insight in whether other indicators of behavioral involvement, such as incorporation of skills in daily life and cooperation of patients during treatment sessions, mediate the association between expectations and treatment outcomes.

The results of the study should be interpreted bearing in mind several strengths and limitations of the study. The strengths of the study are the measurement of homework compliance after every session, multiple assessments of depressive symptoms during treatment, and the inclusion of two different types of treatment. A first limitation of the study is the relatively small sample size per treatment condition. Furthermore, compliance with homework was high on average which may limit the conclusions that can be drawn on the associations between homework compliance and depressive symptom improvement. Future studies might consider manipulating homework experimentally by allocating participants to treatments with varying loads of homework. Also, the extent to which practice is incorporated in daily life could be measured in future studies. Finally, the current study focused specifically on patients with diabetes and depressive symptoms. Caution is warranted when generalizing our findings to other patient populations.

To conclude, the findings suggest that high outcome expectations contribute to treatment completion and positive treatment outcomes, not only in CBT but also in MBCT for depressive symptoms in patients with diabetes. In MBCT, patients may also engage more in homework exercises when they hold high expectations for improvement. The findings imply that the outcomes of CBT and MBCT could be improved by taking the expectations of patients with diabetes into consideration in the choice for type of treatment. The therapeutic process in CBT may be optimized by molding patients’ outcome expectations, for example, by including motivational interviewing as a prelude to treatment (Westra and Dozois 2006). As motivational interviewing does not correspond well with the principles of MBCT (e.g., experiential learning), future research may focus on how patients’ expectations can be optimized in MBCT.
